# The p-Phthalates Terephthalic Acid and Dimethyl Terephthalate Used in the Manufacture of PET Induce In Vitro Adipocytes Dysfunction by Altering Adipogenesis and Thermogenesis Mechanisms

**DOI:** 10.3390/molecules27217645

**Published:** 2022-11-07

**Authors:** Maria Sofia Molonia, Claudia Muscarà, Antonio Speciale, Federica Lina Salamone, Giovanni Toscano, Antonella Saija, Francesco Cimino

**Affiliations:** Department of Chemical, Biological, Pharmaceutical and Environmental Sciences, University of Messina, Viale F. Stagno D’Alcontres 31, 98166 Messina, Italy

**Keywords:** terephthalic acid, dimethyl terephthalate, polyethylene terephthalate, obesogen, endocrine-disrupting chemicals, adipocytes

## Abstract

Public health concerns associated with the potential leaching of substances from Polyethylene terephthalate (PET) packaging have been raised due to the role of phthalates as endocrine-disrupting chemicals or obesogens. In particular, changes in the environment such as pH, temperature, and irradiation can improve contaminant migration from PET food packaging. In this study, the in vitro effects of p-phthalates terephthalic acid (TPA) and dimethyl terephthalate (DMT) on murine adipocytes (3T3-L1) were evaluated using concentrations that might be obtained in adult humans exposed to contaminated sources. TPA and, in particular, DMT exposure during 3T3-L1 differentiation increased the cellular lipid content and induced adipogenic markers PPAR-γ, C/EBPß, FABP4, and FASN, starting from low nanomolar concentrations. Interestingly, the adipogenic action of TPA- and DMT-induced PPAR-γ was reverted by ICI 182,780, a specific antagonist of the estrogen receptor. Furthermore, TPA and DMT affected adipocytes’ thermogenic program, reducing pAMPK and PGC-1α levels, and induced the NF-κB proinflammatory pathway. Given the observed effects of biologically relevant chronic concentrations of these p-phthalates and taking into account humans’ close and constant contact with plastics, it seems appropriate that ascertaining safe levels of TPA and DMT exposure is considered a high priority.

## 1. Introduction

Polyethylene terephthalate (PET) is a thermoplastic polymer broadly used for food and beverage packaging applications, due to gas barrier properties, good thermal and mechanical properties, light weight, transparency, strength, good processability, and good recyclability [1]. PET is produced by polymerizing ethylene glycol with terephthalic acid (TPA) or via transesterification with dimethyl terephthalate (DMT), using antimony trioxide as the catalyst [2]. A more recent development in PET manufacturing is the incorporation of an appropriate comonomer, such as isophthalic acid or DMT, to slow down the rate of crystallization, which allows the manufacture of thicker bottle walls, sheets, and films [3].

Despite the convenience plastic packaging offers to the consumer, it has been subjected to many debates regarding environmental and health issues. Generally, the migration is an undesirable process where polymerization residues or stabilizers can diffuse through the polymer matrix to the surface and then to food. Due to the increasing awareness of consumers in terms of health matters, the importance of the migration of substances from PET food packaging materials to foods attracted the interest of the scientific and legislative communities. According to European Regulation No. 10/2011 [4], the EU addresses the use of phthalates in plastics likely coming into contact with food and beverages and listed certain phthalates (butylbenzyl phthalate (BBP), di [2-ethylhexyl] phthalate (DEHP), dibutyl phthalate (DBP), and diisobutyl phthalate (DIBP)) as toxic for reproduction, so that, starting from January 2015, they were completely banned. European Regulation No. 10/2011 [4] provided a list of authorized substances intentionally used in the manufacture of plastic layers in plastic products indicating specific migration limits (SML) into food or food simulants for these compounds corresponding to the maximum permitted amount that does not present health risks. The background of migration limits considers a conventional assumption that 1 kg of food is consumed everyday by a person of 60 kg body weight and that the food is packaged in a cubic container of 6 dm^2^ surface area releasing the substance. p-Phthalic compounds, belonging to group restriction No 28, include TPA (SML of 7.5 mg/L expressed as mg substance per liter of food simulant and corresponding to 45 µM) and DMT (migration limit of 60 mg/L corresponding to 310 µM). Although these migration limits need to be considered in the risk assessment of the final plastic product, very few data are reported on the impact of biologically relevant concentrations on human health.

Additionally, due to plastic ubiquity in the environment, human exposure to phthalates leached from waste is virtually unavoidable [5].

In recent years, the intake of phthalates-contaminated food and drinking water has been considered the major route of human exposure to these plasticizers [6], accounting for more than 67% [7]. Casajuana and Lacorte [8] analyzed PET bottles of mineral water of different trades. Phthalates were present at very low initial concentrations in water, whereas they found increased concentrations after storing PET bottles for 10 weeks at up to 30 °C. In addition, different conditions such as pH, storage time, storage temperature (30–60 °C), and exposure to sunlight may influence the phthalate concentrations in PET bottled water [9].

Epidemiologic studies found that early phthalates exposure could induce significant neuro-developmental damage and is suspected to cause endocrine-disrupting effects in humans [10]. In addition, the obesogenic activity of these compounds has been reported [11]. According to Buser et al., phthalate metabolites are able to cause obesity in male children and adolescents and contribute to obesity in adults [12]. In a Chinese study, children’s exposure to mono (2-ethylhexyl) phthalate (MEHP) elicited an increase in the body mass index and waist circumference [13].

The mode of action underlying phthalate toxicity remains unclear. However, it was proposed that peroxisome proliferator-activated receptors (PPARs) may be involved in obesity following phthalate exposure because many phthalate monoesters, including MEHP and mono-isodecyl phthalate (MDP), were found to induce PPARs in vitro [14,15,16]. As PPARs are known to be key players in lipid and glucose homeostasis [17], it is reasonable that phthalates’ involvement in metabolic disorders likely correlates with their binding to PPAR-α and γ, the latter associated with adipogenesis and controlled by neuroendocrine pathways involved in the hypothalamic–pituitary–adrenal axis [18], but also to other receptors such as steroid hormone receptors, thyroid hormone receptors, retinoid X receptors, liver X receptors, and farnesoid X receptors [19]. Additionally, there is recent evidence showing that phthalates exert obesogenic activity by affecting the production or function of thermogenic adipocytes involved in energy consumption instead of accumulation as triglycerides [20].

However, data reported in the literature focused mainly on ortho-phthalates. Compared to these, there is a general lack of information for the other isomers, meta- and para-phthalates (or p-phthalates), except for diethyl hexyl terephthalate (DEHT) [21,22]. However, due to the structural similarity to ortho-phthalates, it is very important to assess the metabolic activities of other phthalate isomers since they can occur at environmentally relevant, although low, concentrations and chronic exposure to such concentrations could have a significant impact on human health.

In this paper, we intended to evaluate, for the first time, the effects of the p-phthalates TPA and DMT on adipose tissue using concentrations mimicking those that might be obtained in adult humans through the consumption of foods in PET packages or exposure to other contaminated sources [23,24]. Furthermore, we tested TPA and DMT at 10 nM, taking into consideration the environmentally relevant concentrations of phthalates as measured in the blood of human subjects [25,26]. Of course, the doses used in other studies may be relevant in the case of other routes of exposure to p-phthalates, such as inhalation or cutaneous absorption, or also if these compounds are hypothesized to be ingested through products other than food, for example, dust [27,28]. With this aim, 3T3-L1 adipocytes were selected as an in vitro model to clarify the potential obesogenic effects of these phthalate isomers, evaluating the adipogenesis process and tissue inflammation and the potential mechanisms of the observed effects, and thus providing an experimental basis for the correlation between p-phthalates and obesity.

## 2. Results

### 2.1. p-Phthalates In Vitro Toxicity and In Silico ADME

**Figure 1:**
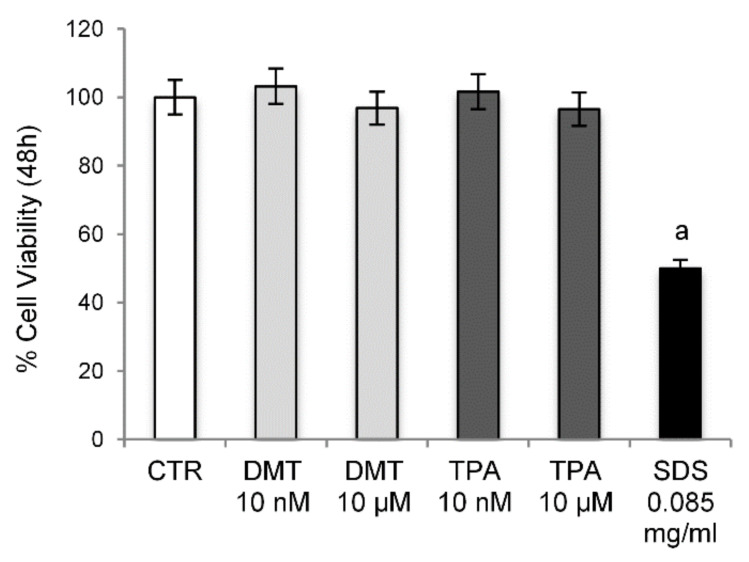
Cell Viability. Cytotoxicity was evaluated by sulforhodamine B assay on NIH/3T3 cells treated with different concentrations of the tested compounds for 48 h. Control cells were treated with the vehicle (DMSO) alone. SDS was used as a positive control. ^a^ *p* < 0.05 vs. CTR.

The cytotoxic potential of DMT and TPA (10 nM and 10 µM) has been investigated by sulforhodamine B assay on NIH/3T3 mouse fibroblasts, after 48 h of exposure. The results obtained showed that the tested p-phthalates do not have any cytotoxic effect (Figure 1).

As reported in Table 1, the tested compounds followed Lipinski’s rules for drug-likeness with no violation. Regarding the in silico ADME analysis, the results may be interpreted based on the marginal value compared with the resultant value as follows: High Caco-2 permeability is predicted by a value > 0.90, and intestinal absorption less than 30% is considered poorly absorbed; human VDss is low if below 0.71 L/kg and high if above 2.81 L/kg. In particular, both tested compounds appear to possess good oral bioavailability. The percentage of intestinal absorption of all the compounds has been calculated with values ranging from 75% (TPA) to 90% (DMT) and confirmed with Caco-2 permeability.

### 2.2. Effects of p-Phthalates on Adipogenesis Markers

**Figure 2:**
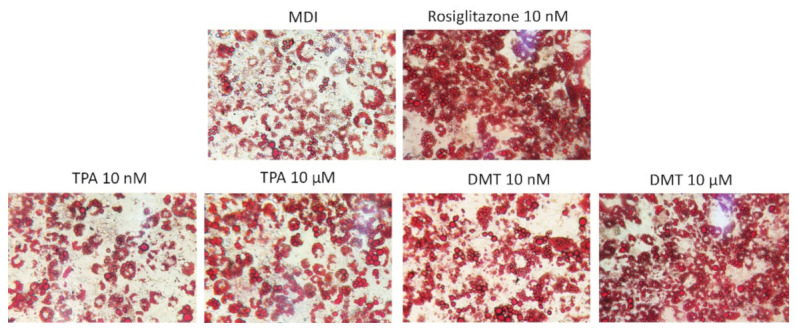
Oil Red O staining. 3T3-L1 preadipocytes were cultured in differentiation medium (MDI) containing phthalates (10 nM and 10 μM) or rosiglitazone (10 nM, positive control) for 10 days. Cells treated with MDI alone were used as controls. The cells were then stained with Oil Red O and photographed using an optical microscope (original magnification at ×40). The images are representative of three independent experiments.

Phthalates appear to play an essential role in the development of obesity, interfering at the level of the expression of key transcription factors in the adipogenesis process, such as SREBP-1c, PPAR-γ, C/EBPα, and LXRα, inducing important metabolic alterations [29]. Their activation determines, indeed, the upregulation of lipogenic genes resulting in the accumulation of intracellular lipids and adipocyte hypertrophy [30]. Therefore, in order to determine the effects of terephthalic acid and its dimethyl ester on adipocytes’ differentiation, the 3T3-L1 preadipocytes were exposed to the tested compounds for 10 days, and the accumulation of intracellular lipids was evaluated by the histological technique of Oil Red O staining. The established adipogenesis activator rosiglitazone, a thiazolidinedione agonist of the PPAR-γ receptor, was used as the positive control. The results obtained showed that, under our experimental conditions, the treatment with the tested phthalates causes an increase in the size of lipid droplets compared to control cells in a dose-dependent manner. In particular, the extent of lipid accumulation induced by TPA was 1.27 ± 0.03 and 3.75 ± 0.05 vs. MDI (for 10 nM and 10 μM, respectively) and by DMT was 4.25 ± 0.03 and 5.04 ± 0.02 vs. MDI (for 10 nM and 10 μM, respectively) (Figure 2). As expected, rosiglitazone-exposed cells showed strong enhancement (approximately 5.22-fold) in lipid accumulation compared to untreated cells.

**Figure 3:**
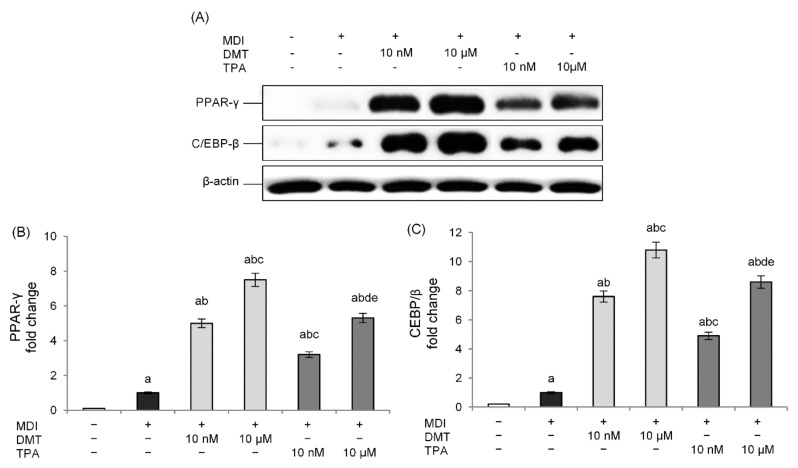
Effects of p-phthalates on PPAR-γ (**A**,**B**) and CEBP/β (**A**,**C**) expression. 3T3–L1 preadipocytes were cultured in differentiation medium (MDI) containing p-phthalates (10 nM and 10 μM) for 10 days. Cells treated with MDI alone were used as controls. Undifferentiated cells were cultured in standard medium for 10 days. The densitometry results (**B**,**C**) are reported as fold change compared to MDI. The intensity values of the PPAR-γ and CEBP/β protein were normalized to the corresponding values of β-actin. All data are expressed as mean ± SD of three independent experiments. ^a^ *p* < 0.05 vs. undifferentiated cells; ^b^ *p* < 0.05 vs. MDI; ^c^ *p* < 0.05 vs. DMT 10 nM; ^d^ *p* < 0.05 vs. DMT 10 µM; ^e^ *p* < 0.05 vs. TPA 10 nM.

Although the exact mechanism of action remains unclear, several studies have shown how the PPARs may be involved in obesity following ortho-phthalate exposure [15,31,32]. These transcriptional factors regulate, indeed, a broad range of physiological processes including adipogenesis, fatty acid uptake, and cell proliferation, and are essential for the metabolism of lipids [33]. Therefore, in order to evaluate the mechanism through which p-phthalates determine the accumulation of lipid deposits, the expression of PPAR-γ was evaluated. The obtained results confirm that exposure to the tested phthalates induces a marked increase in PPAR-γ levels, with respect to unexposed cells, in a dose-dependent manner (Figure 3A,B). In particular, low nanomolar levels were already able to activate this transcription factor, and the rank order of PPAR-γ induction was DMT > TPA.

We also examined the level of expression of C/EBP-β, which is an important transcription factor expressed at the early stage of adipocyte differentiation, induced rapidly after the addition of adipogenic stimuli, and responsible for stimulating the expression of PPAR-γ [34]. The obtained results show a dose-dependent increase in C/EBP-β expression with respect to unexposed differentiated cells following treatment with the tested p-phthalates. Additionally, in this case, DMT showed higher activity compared to TPA. These data demonstrate how the effects of these substances in the induction of the adipogenesis process occur from the early stage of adipocyte differentiation (Figure 3A,C).

**Figure 4:**
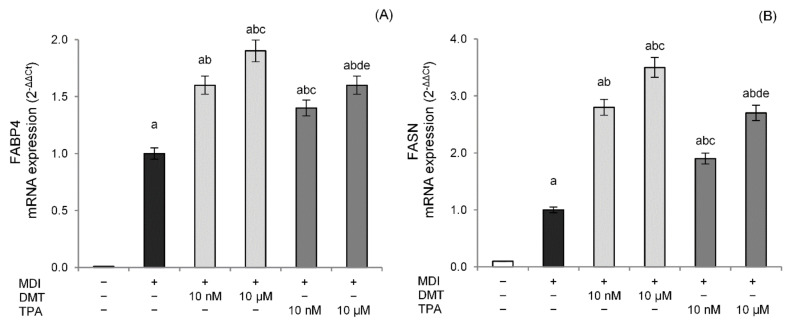
Effect of p-phthalates on FAPB4 (**A**) and FASN (**B**) mRNA expression. 3T3–L1 preadipocytes were cultured in differentiation medium (MDI) containing p-phthalates (10 nM and 10 μM) for 10 days. Cells treated with MDI alone were used as controls. Undifferentiated cells were cultured in standard medium for 10 days. FAPB4 and FASN mRNA expression was analyzed by real-time PCR and data are expressed as 2^−ΔΔCt^ and normalized to MDI. 18S rRNA was used as housekeeping gene. All data are expressed as mean ± SD of three independent experiments. ^a^ *p* < 0.05 vs. undifferentiated cells; ^b^ *p* < 0.05 vs. MDI; ^c^ *p* < 0.05 vs. DMT 10 nM; ^d^ *p* < 0.05 vs. DMT 10 µM; ^e^ *p* < 0.05 vs. TPA 10 nM.

As further confirmation of the adipogenic effect induced by p-phthalates, we evaluated the gene expression of FABP4, a downstream gene of the PPAR-γ signaling pathway and a key regulator of fatty acid uptake and lipid accumulation during the adipogenic process [35]. FABP4-deficient adipocytes exhibit, in fact, reduced efficiency in adipogenesis and lipogenesis [36]. The data shown in Figure 4A also demonstrate how p-phthalates, already at nanomolar levels, are able to induce FABP4 gene expression in comparison to unexposed differentiated cells, confirming the activation of the PPAR-γ pathway. DMT-exposed cells showed higher transcriptional activity compared to those exposed to TPA. Furthermore, the expression of the gene-encoding fatty acid synthase (FASN), a central enzyme involved in the lipogenesis process since it catalyzes the de novo biosynthesis of long-chain saturated fatty acids in adipose tissue, was evaluated. The tested p-phthalates significantly induced a dose-dependent increase in FASN gene expression compared to unexposed differentiated cells (Figure 4B), thus further suggesting the important role of these compounds in lipogenic pathway activation.

### 2.3. p-Phthalates-Induced PPAR-γ Expression Is Dependent on Estrogen Receptor (ER)

**Figure 5:**
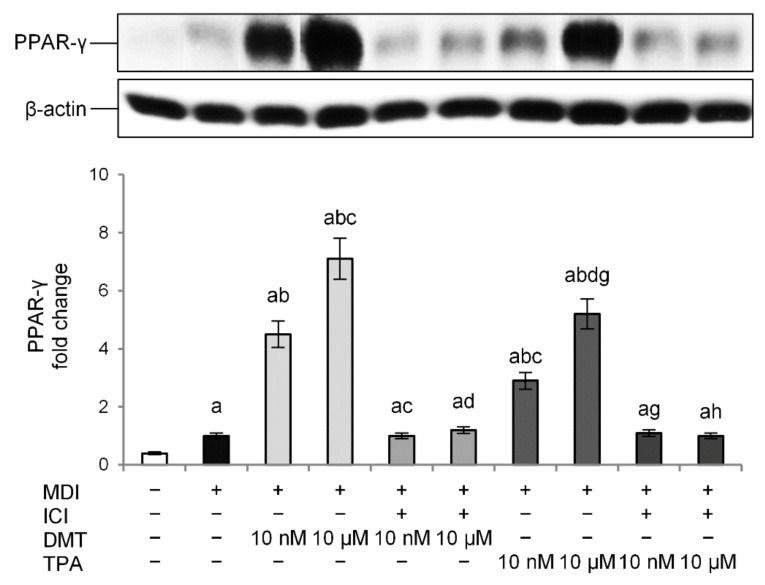
Effect of the ER antagonist ICI on the obesogenic effect of p-phthalates: PPAR-γ expression. 3T3–L1 preadipocytes were cultured in differentiation medium (MDI) containing p-phthalates (10 nM and 10 μM) and ICI 10 nM for 10 days. Cells treated with MDI alone were used as controls. Undifferentiated cells were cultured in standard medium for 10 days. The densitometry results are reported as fold change compared to MDI. The intensity values of the PPAR-γ were normalized to the corresponding value of β-actin. All data are expressed as mean ± SD of three independent experiments. ^a^ *p* < 0.05 vs. undifferentiated cells; ^b^ *p* < 0.05 vs. MDI; ^c^ *p* < 0.05 vs. DMT 10 nM; ^d^ *p* < 0.05 vs. DMT 10 µM; ^g^ *p* < 0.05 vs. TPA 10 nM; ^h^ *p* < 0.05 vs. TPA 10 µM.

To further demonstrate whether p-phthalates-induced effects on preadipocytes’ differentiation are mediated through the ER, adipocytes were cotreated with the tested compounds (10 nM or 10 µM) and the ER antagonist ICI (10 nM) during differentiation. ER antagonists provide a good tool to study the effect of a lack of estrogen action on the target tissue. In our experimental conditions, treatment with ICI alone did not affect the basal levels of PPAR-γ (data not shown). On the contrary, the ER antagonist prevented TPA- and DMT-induced protein expression, restoring PPAR-γ levels to that of the control (Figure 5). Interestingly, PPAR-γ expression was inhibited with both 10 nM and 10 µM concentrations of TPA and DMT. These data suggest that the mechanism of p-phthalates-induced differentiation of 3T3-L1 preadipocytes may involve an ER-mediated mechanism.

### 2.4. p-Phthalates Reduce the Thermogenic Pathway

Recent insights into adipocyte function recognized the potential to contribute to energy turnover through thermogenic brown adipose tissue (BAT), which is specialized in dissipated energy compared to white adipose tissue (WAT), which accumulates it as triglycerides. However, WAT is also able to express a “brown-like” phenotype promoting mitochondrial biogenesis modulated by the AMP-activated protein kinase (AMPK) pathway, a crucial energy sensor that regulates energy metabolism by activating PGC-1α [37]. Some evidence showed that obesogens are associated with the disruption of thermogenic fat and adipose tissue, and these effects are related to the altered AMPK pathway [20,37]. Reduced thermogenesis is associated with lower energy expenditure, higher adiposity, and increased risk of insulin resistance [38].

**Figure 6:**
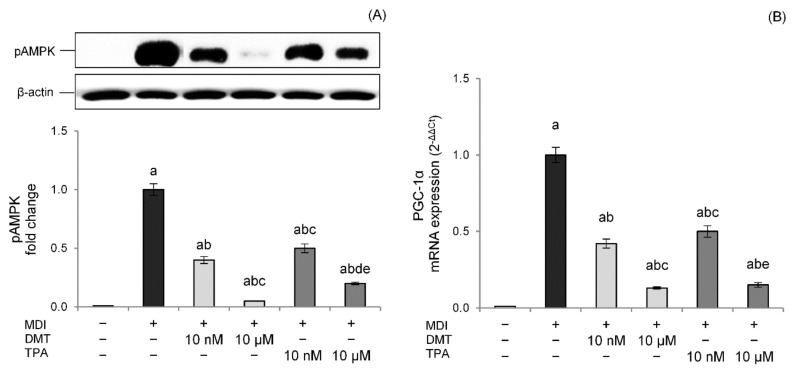
Effects of p-phthalates on pAMPK expression (**A**) and PGC1α gene expression (**B**). 3T3–L1 preadipocytes were cultured in differentiation medium (MDI) containing p-phthalates (10 nM and 10 μM) for 10 days. Cells treated with MDI alone were used as controls. Undifferentiated cells were cultured in standard medium for 10 days. The densitometry results are reported as fold change compared to MDI. The intensity values of the pAMPK protein were normalized to the corresponding value of β-actin (**A**). PGC1α gene expression values (**B**) are expressed as 2^−ΔΔCt^ and normalized against MDI. 18S rRNA was used as housekeeping gene. All data are expressed as mean ± SD of three independent experiments. ^a^ *p* < 0.05 vs. undifferentiated cells; ^b^ *p* < 0.05 vs. MDI; ^c^ *p* < 0.05 vs. DMT 10 nM; ^d^ *p* < 0.05 vs. DMT 10 µM; ^e^ *p* < 0.05 vs. TPA 10 nM.

To confirm and further extend these findings, we examined the effects of p-phthalates exposure on thermogenesis during adipogenesis differentiation. The results showed that, in our experimental conditions, DMT and TPA reduced AMPK levels in a dose-dependent way compared to unexposed differentiated control cells (Figure 6A). Interestingly, these effects were also evident at nanomolar concentrations, with DMT being more effective than TPA in AMPK inhibition.

Thermogenesis activated by AMPK relies on PGC-1α, a cAMP-dependent protein that promotes mitochondrial biogenesis and oxidative metabolism [39]. PGC-1α coactivates different nuclear receptors involved in the transcriptional induction of markers of thermogenesis such as UCP-1 [40]. Data confirmed lower PGC-1α mRNA levels in DMT- and TPA-treated cells with a dose-dependent effect, corroborating the impaired thermogenesis pathway (Figure 6B).

### 2.5. p-Phthalates Induce the NF-κB Proinflammatory Pathway

**Figure 7:**
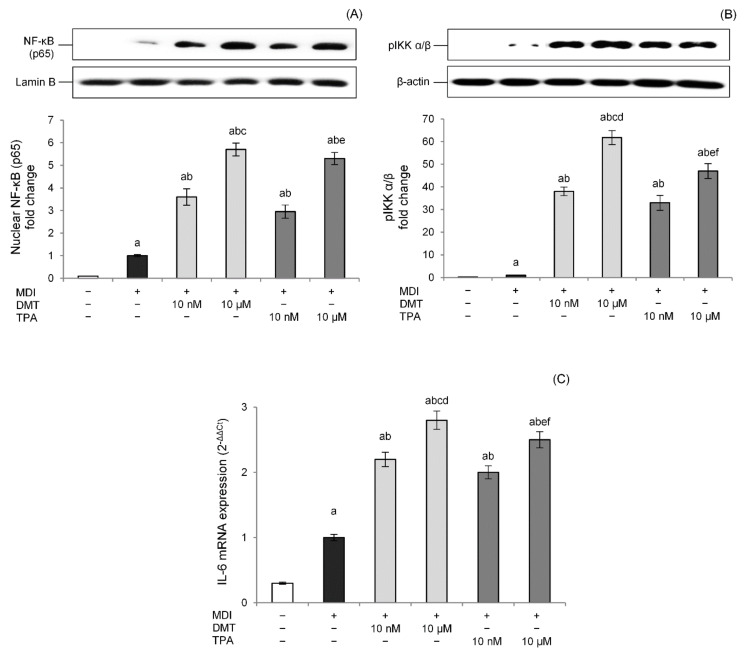
Effects of p-phthalates on nuclear levels of NF-κB (p65) (**A**), cytosolic levels of p-IKK α/β (**B**), and IL-6 gene expression (**C**). 3T3–L1 preadipocytes were cultured in differentiation medium (MDI) containing p-phthalates (10 nM and 10 μM) for 10 days. Cells treated with MDI alone were used as controls. Undifferentiated cells were cultured in standard medium for 10 days. Nuclear (**A**) and cytosolic (**B**) proteins were analyzed by western blot. Results by densitometry are reported as fold change against CTR and expressed as mean ± SD of three independent experiments. NF-κB (p65) intensity values were normalized to the corresponding Lamin B values (**A**), whereas p-IKK α/β intensity values were normalized to the corresponding β-actin values (**B**). IL-6 gene expression values (**C**) are expressed as 2^−ΔΔCt^ and normalized against control (MDI). 18S rRNA was used as housekeeping gene. ^a^ *p* < 0.05 vs. undifferentiated cells; ^b^ *p* < 0.05 vs. MDI; ^c^ *p* < 0.05 vs. DMT 10 nM; ^d^ *p* < 0.05 vs. all TPA concentrations; ^e^ *p* < 0.05 vs. TPA 10 nM; ^f^ *p* < 0.05 vs. all DMT concentrations.

Excessive storage of fatty acids in adipose tissue produces hypertrophic and dysfunctional adipocytes, thereby secreting several proinflammatory adipokines such as IL-6, IL-8, IFN-γ, and TNF-α and inducing a state of chronic low-grade inflammation [41]. In particular, nuclear factor (NF)-κB, the main transcriptional factor involved in inflammatory processes [42], plays a central role in inflammation-mediated metabolic disorders. Therefore, to evaluate the effects of p-phthalates on adipocytes’ inflammation, the NF-κB pathway was studied. Data showed that p-phthalates induce NF-κB p65 nuclear translocation in a dose-dependent way (Figure 7A). Interestingly, low nanomolar concentrations were already able to activate the NF-κB pathway, and this effect was more evident for DMT.

The canonical pathway of NF-κB is triggered through the enzymatic activity of an IκB kinase (IKK), which induces the disengagement of the IκB inhibitor from NF-κB, allowing its translocation to the cell nucleus. p-Phthalates dose-dependently induced pIKK levels, confirming that these compounds modulate the NF-κB pathway, improving the phosphorylation of the IκB inhibitor by IKK. Additionally, in this case, the effect was more prominent for DMT compared to TPA (Figure 7B). Additionally, the transcriptional activity of the NF-κB pathway was studied evaluating IL-6 gene expression, an NF-κB-regulated cytokine connected to the activation of chronic inflammatory pathways in obese people [43]. Results showed that DMT and TPA induce IL-6 gene expression at low nanomolar concentrations, and this effect was dose-dependent (Figure 7C). Moreover, in this case, DMT 10 µM was able to induce NF-κB transcriptional activity better than TPA (10 µM).

## 3. Discussion

PET is the most common plastic for thermoforming packaging because of its low cost, physical and chemical tolerance, and easy recycling procedure. PET is produced starting with the esterification of TPA or transesterification of DMT with ethylene glycol to produce monomers, followed by polycondensation in the presence of a catalyst. However, public health concerns associated with the potential leaching of substances from PET packaging have been raised due to their role as endocrine-disrupting obesogens. Phthalates can be released from plastic products into the environment during their manufacture, storage, use, or disposal, inducing exposure and accumulation in humans. In particular, the main source of human exposure is the consumption of food that has been in contact with containers and products containing phthalates or through other sources contaminated by PET wastes [23]. Phthalates have been demonstrated to induce negative effects on human health and are suspected carcinogens, as well as toxic to the liver, kidneys, and reproductive organs [44]. Additionally, phthalates have been considered potential endocrine-disrupting substances since they are able to interfere with hormone receptors, disrupting the homeostasis system in the body and interfering with regulatory processes in metabolism and in the control of adipocyte function [45].

Ortho-phthalates, generally known as phthalates, are one of the three isomeric forms of benzenedicarboxylic acid and are considered the most biologically active phthalates. There is a general lack of information in the scientific literature on p-phthalates (or terephthalates), with the exception of DEHT, generally supporting a different and lower mammalian toxicity profile compared to other isomers [46,47]. In vitro nanomolar TPA exposure induced cellular perturbations evident as the induction of DNA damage, cell cycle arrest, and subsequent escape from programmed cell death in non-malignant human high-risk donor breast epithelial cells (HRBECs) isolated from several donors [48]. Additionally, DMT inhibited in vitro WiL2-NS human B cell proliferation, likely by enhancing ROS production [49].

In our study, due to the similar structure between phthalate isomers, we hypothesized that p-phthalates can affect fat metabolism and adipogenesis at environmentally relevant low concentrations. With this aim, we evaluated adipogenic activity in differentiation experiments with murine preadipocyte 3T3-L1 cells.

In our experimental model, cells were exposed to TPA and DMT at levels mimicking those that might be obtained in adult humans with the consumption of foods contained in PET packages, similar to other findings reported in the literature [50,51] or environmentally relevant concentrations found in the blood of human subjects [25,26]. One has to take into consideration that intestinal absorption of 100% of the phthalates after oral ingestion may be hypothesized; as said before, the in silico ADME analysis showed intestinal absorption of p-phthalates ranging between 75% (TPA) and 90% (DMT) (Table 1).

Our data showed that DMT and TPA exposure during 3T3-L1 differentiation dose-dependently increased the lipid content compared to unexposed differentiated cells. Although we observed a significative effect starting from 10 nM, DMT 10 μM induced the maximal lipid droplet formation, similar to that of the positive control rosiglitazone. Furthermore, among the p-phthalates tested, DMT was more effective than TPA. Since DMT shows better lipophilic features than those of TPA, this could explain, at least partially, why the effects of DMT were more marked than that induced by TPA. In fact, one hypothesis is that DMT could easily be transported across lipidic membranes and reach intracellular targets. The molecular mechanisms controlling adipogenesis in the 3T3-L1 cells involve the expression of key adipogenic transcription factors. In particular, the expression of the transcription factor PPAR-γ is required for adipocyte formation and maturation, upregulating the expression of proteins that play a role in lipid metabolism, such as FABP4 and FASN. The cytoplasmic protein FABP4 binds long-chain fatty acids and other hydrophobic ligands and is involved in fatty acid uptake, transport, and metabolism. FASN is critical in the regulation of body weight since it is required for de novo synthesis of long-chain saturated fatty acids from acetyl coenzyme A (CoA), malonyl-CoA, and NADPH [52]. At the molecular level, DMT and TPA induced PPAR-γ in a dose-dependent way, confirming the effects of p-phthalates in the adipogenic process. To the same extent, p-phthalates activated PPAR-γ transcriptional machinery inducing FABP4 and FASN gene expression. However, p-phthalates increased C/EBPβ expression, a transcription factor acting during the induction of 3T3-L1 differentiation. Since C/EBPβ is required for binding to DNA adipogenic hotspots of other adipogenic transcription factors and is an inducer of PPAR-γ transcription, we can speculate that the effects of p-phthalates are not due to direct PPAR-γ activation but rather to the modulation of early events in preadipocytes’ differentiation. This mechanism was also proposed for the ortho-phthalate DEHP, since relative mRNA and protein levels of CEBP/β in adipose tissue of rats exposed to DEHP were significantly higher than in the control group [53]. Interestingly, the adipogenic action of TPA- and DMT-induced PPAR-γ was reverted by ICI, a specific antagonist of the ER, suggesting that p-phthalates likely induce adipocyte differentiation through an ER-mediated mechanism. Phthalates, in fact, have been demonstrated to induce estrogenic effects [54,55] modulating ERα or ERß activities [56], even if few data are reported for these compounds. Additionally, adipogenesis induced by bisphenol A (BPA), a well-known endocrine disruptor, was reverted by ICI in 3T3-L1 adipocytes, although the involvement of other non-classical ER pathways was suggested [57]. However, our data, although preliminary, need further confirmation using specific in vitro and cell-based assays able to study direct or indirect ER targeting [58].

As part of the obesity phenotype, dysfunctional adipocytes show increased stores of energy such as triglycerides in unilocular lipid droplets, instead of dissipating it as heat through the activation of the thermogenic program. In particular, AMPK plays an important role in WATm inhibiting fatty acid synthesis and promoting fatty acid oxidation via thermogenic processes inducing a “brown-like” phenotype [37]. Our data demonstrated the dose-dependent inhibitory effects of DMT and TPA on AMPK phosphorylation at all the concentrations used. Interestingly, lower nanomolar concentrations were already able to significantly reduce basal pAMPK levels. The effects on the negative imbalance of AMPK exerted by p-phthalates were confirmed by the inhibition of PGC-1α, a cAMP-dependent protein able to induce mitochondrial biogenesis and dissipate lipids to generate heat, and these results support the hypothesis of a reduced “brown-like” phenotype in 3T3-L1. Our data find support from the literature since dibutyl phthalate inhibits the phosphorylation of AMPK in the liver, significantly promotes liver enlargement, and increases triglyceride and cholesterol levels in Sprague–Dawley rats [59]. Furthermore, DEHP reduced the AMPK downstream phosphorylation of ACC2 in human SGBS-adipocytes, the key enzyme of lipogenesis, improving fatty acid beta-oxidation [60]. Additionally, obesogen BPA reduced pAMPK and its downstream enzyme pACC levels in 3T3-L1 cells [61].

Obesity is characterized by a state of chronic inflammation in adipose tissue mediated by the secretion of a range of inflammatory cytokines. There is increasing evidence that inflammation affects the thermogenic activity of adipose tissue by impairing its capacity for energy expenditure, contributing to adipocytes’ dysfunction in obesity [62]. Herein, we observed that DMT and TPA dose-dependently induced NF-κB pathway activation at low nanomolar levels with slight differences between the two compounds at 10 µM. The promotion of an inflammatory state in adipocytes has also been demonstrated for other phthalates and obesogens at nanomolar concentrations [63]. DEHP exposure was also shown to induce rat adipose tissue infiltration with macrophages with the subsequent secretion of TNF-α and IL-1b, which promoted adipose tissue dysfunction and altered lipid metabolism [53]. In agreement, in utero DEHP exposure in Sprague–Dawley rats induced both adipose tissue and systemic inflammation in parallel with increasing preadipocyte differentiation [64]. It is also proposed that PPAR-γ may be at least partially responsible for the proinflammatory response in differentiated murine adipocytes in parallel with the adipogenic effects [63].

## 4. Materials and Methods

### 4.1. In Silico ADME Screening of Tested Compounds

The molecules were screened using the online tool (http://biosig.unimelb.edu.au/pkcsm/prediction accessed on 5 April 2022) to predict their important pharmacokinetic properties. ADME (absorption, distribution, metabolism, and excretion) properties include absorption, Caco-2 permeability, water solubility, human intestinal absorption, P-glycoprotein substrate, P-glycoprotein I and II inhibitors, skin permeability, distribution, steady-state volume of distribution (VDss), and fraction unbound [65].

The drug-likeness properties were screened using the online tool Molinspiration (https://www.molinspiration.com/cgi-bin/properties accessed on 5 April 2022) based on the Lipinski Rules of five [66]. Molinspiration supports the calculation of important molecular properties such as Log P, polar surface area, and the number of hydrogen bond donors and acceptors. The calculation of Log P is based on a formula considering the lipophilicity, hydrophobicity, and polarity of the compound [67].

The Rule of Five (or Lipinski’s rule), stated for compounds that are not substrates for active transporters, is able to predict the absorption or permeation of a potential drug candidate by combining specific parameters [68]. According to this, poor oral bioavailability is more likely when there are more than 5 H-bond donors, 10 H-bond acceptors, the molecular weight is greater than 500, and the calculated Log P is greater than 5. In general, an orally active drug has no more than one violation of these criteria.

### 4.2. Reagents

Dimethyl sulfoxide (DMSO), methanol, and ethanol were obtained from Carlo Erba Reagent (Milan, Italy) in their highest commercially available purity grade. DMT (code #31298-250MG), TPA (code #40818-100MG), and all other reagents, if not differently specified, were purchased from Merk Life Science (Milan, Italy).

### 4.3. Cell Culture and Treatments

The 3T3-L1 murine preadipocyte cells, obtained from the American Tissue Culture Collection (Manassas, VA, USA), were grown in Dulbecco’s modified essential medium (DMEM) supplemented with 10% newborn calf serum, 4 mM L-glutamine, 100 U/mL penicillin/streptomycin solution, and 25-mM HEPES buffer. Cells were maintained at 37 °C in an incubator with a humidified atmosphere containing 95% air and 5% CO_2_. To prepare 3T3-L1 monolayers, cells were plated at 1.3 × 10^4^ cells/cm^2^ in multiwell plates and cultured for 10 days after confluence in a differentiation medium to obtain totally differentiated cells, morphologically similar to white adipocytes. In particular, the cells were incubated for 4 days with DMEM supplemented with 10% fetal bovine serum, 4 mM L-glutamine, 100 U/mL penicillin/streptomycin solution, and 25-mM HEPES buffer, containing prodifferentiating agents (MDI: 0.5 mM 3-isobutyl-1-methylxanthine, 1 μΜ dexamethasone, and 1 μg/mL insulin). The cells were then maintained in DMEM supplemented with fetal bovine serum (see above) containing only 1 μg/mL insulin for the following 6 days, up to the total differentiation in mature adipocytes. In this phase, the medium was replicated every 3 days. The cells were always used within the 20th passage because differentiation efficiency declines rapidly with higher passage numbers.

For all the experiments, 3T3-L1 preadipocytes were treated, throughout all 10 days of the differentiation process described above, with DMT and TPA at two different concentrations (10 nM and 10 µM). The tested p-phthalates were always freshly dissolved in DMSO and immediately used. Rosiglitazone (10 nM) was used as the positive control. The final concentration of DMSO in the culture medium during the different treatments was ≤0.1% (*v*/*v*), while the cells treated with the vehicle (DMSO 0.1% *v*/*v*) and then exposed only to the differentiation inducers were used as controls. At the end of the exposure time, cells were immediately processed and/or preserved at −80 °C until the analysis required for each test.

For the ER antagonist studies, 10 nM ICI-182,780 (ICI) was added during all differentiation periods in the presence or not of DMT and TPA (10 nM and 10 µM).

For the toxicity test, the murine fibroblast cell line NIH/3T3 (ATCC, Rockville, MD, USA) was employed. Cells were cultured in DMEM supplemented with 10% fetal bovine serum, 4 mM L-glutamine, and a 100 U/mL penicillin/streptomycin solution, and maintained in an incubator with a humidified atmosphere containing 5% CO_2_ at 37 °C. In the experiments, cells were plated in 24-well cell plates at an initial density of 9 × 10^4^ cells/well, and after 24 h, semi-confluent monolayers were treated for 48 h with DMT and TPA (10 nM and 10 µM) added to the cell culture medium. Sodium dodecyl sulfate (SDS) was used as a positive control at the historical mean concentration established by the laboratory (0.085 mg/mL). The cells treated with the vehicle alone (DMSO 0.1% *v*/*v*) were used as controls.

### 4.4. Sulforhodamine B Assay

The cytotoxic effect of TPA and DMT on NIH/3T3 cells was evaluated using the sulforhodamine B assay, based on the measurement of cellular protein content according to Anwar et al. [69]. In detail, following treatment, the cells were fixed for 1 h at 4 °C using 10% trichloroacetic acid (*w*/*v*). After this step, cells were washed twice with distilled water and incubated for 30 min at room temperature with sulforhodamine B (0.4% *w*/*v* in 1% acetic acid). Then the excess dye was removed by washing repeatedly with 1% (*v*/*v*) acetic acid, and the dye trapped in the cells was solubilized in a 10 mM Tris base solution. Therefore, the absorbance was measured at 565 nm using a microplate reader.

### 4.5. Cell Lysates Preparation

Following the appropriate treatments, 3T3-L1 cells were washed with DPBS and harvested with a scraper. For total and nuclear lysates’ preparation, the cells were resuspended in a lysis buffer containing protease inhibitors and dithiothreitol (DTT) as previously described [70].

After centrifugation, the protein fraction was stored at −20 °C until use. The protein concentration in lysates was assessed using the Bradford assay [71].

### 4.6. Immunoblotting

For immunoblot analyses, 20 μg of nuclear lysates or 30 μg of total or cytosolic lysates were denatured in 4 × SDS-PAGE reducing sample buffer (260 mM Tris–HCl, pH 8.0, 40% (*v*/*v*) glycerol, 9.2% (*w*/*v*) SDS, 0.04% bromo-phenol blue, and 2-mercaptoethanol as reducing agent) and subjected to SDS-PAGE on 10% acrylamide/bisacrylamide gels. Following the separation, the proteins were transferred to Polyvinylidene Difluoride (PVDF) membranes (Hybond-P PVDF, Amersham Bioscience, Milan, Italy). Residual binding sites on the membrane were blocked for 1 h at room temperature with 5% lyophilized non-fat milk solubilized (*w*/*v* in TBST: 10 mM Tris-base, 100 mM NaCl and 0.1% Tween 20).

Membranes were then incubated overnight with specific primary antibodies: Mouse anti-PPAR-γ monoclonal antibody (Santa Cruz Biotechnology, Dallas, TX, USA) (1:1500), rabbit anti-NF-κB p65 polyclonal antibody (Invitrogen) (1:1000), rabbit anti-Phospho-IKK α/β (Ser176/180) monoclonal antibody (Cell Signaling Technology, Danvers, MA, USA) (1:1000), rabbit anti-pAMPK monoclonal antibody (Cell Signaling Technology, Danvers, MA, USA) (1:1000), rabbit anti C/EBPβ polyclonal antibody (Cell Signaling Technology, Danvers, MA, USA) (1:1000), rabbit anti-β-Actin monoclonal antibody (Cell Signaling Technology, Danvers, MA, USA) (1:6000), and rabbit anti-Lamin-B monoclonal antibody (Cell Signaling Technology, Danvers, MA, USA) (1:1500), followed by 2 h incubation with peroxidase-conjugated secondary antibody HRP labeled goat antirabbit Ig (Cell Signaling Technolog, Danvers, MA, USA) (1:6000), goat anti-Mouse IgM Secondary Antibody, and HRP conjugate (Cell Signaling Technology, Danvers, MA, USA) (1:6000), and visualized with an ECL plus detection system (Amersham Biosciences, Milan, Italy). Quantitative analysis was performed by densitometry. The equivalent loading of proteins in each well was confirmed by Ponceau staining and a β-actin or Lamin B control.

### 4.7. Real-Time PCR

Total RNA was extracted using the E.Z.N.A.^®^ Total RNA kit according to the manufacturer’s instruction (OMEGA Bio-Tek, VWR, Milan, Italy), quantified by the Quanti-iT TM RNA assay kit QUBIT (Invitrogen, Milan, Italy), and reverse transcribed with M-MLV Reverse Transcriptase. A quantitative real-time polymerase chain reaction (PCR; Applied Biosystems 7300 Real-Time PCR System, Foster City, CA, USA) coupled with SYBR green chemistry (SYBR green JumpStart Taq Ready Mix) was performed for the identification of mRNA levels of IL6 (FW 5′-GATGGATGCTACCAAACTGGAT-3′, RV 5′-CCAGGTAGCTATGGTACTCCAGA-3′ [72]), FABP4 (FW 5′-AAGGTGAAGAGCAT CATAACCCT-3′, RV 5′-TCACGCCTTTCATAACACATTCC-3′ [73]), FASN (FW 5′-GGA GGT GGT GAT AGC CGG TAT-3′, RV 5′-TGG GTA ATC CAT AGA GCC CAG-3′ [74]), PGC-1α (FW 5′-TGTGGAACTCTCTGGAACTGC-3′, RV 5′-GCCTTGAAAGGGTTATCTTGG-3′ [75]), and 18S rRNA (FW 5′-GTAACCCGTTGAACCCCATT-3′, RV 5′-CCATCCAATCGGTAGTAGCG-3′ [73]) was used as reference gene. For IL6 and 18S amplification, the following parameters were applied: 40 cycles of 94 °C denaturation (15 s), 60 °C annealing, and extension (60 s). FABP4 and FASN were instead amplified using 40 cycles of 95 °C denaturation (45 s), 60 °C annealing, and extension (60 s). Data were elaborated by SDS 1.3.1 software (Applied Biosystems, Foster City, CA, USA) and expressed as the threshold cycle (Ct). The fold increase in mRNA expression compared with the control cells not pre-treated and not exposed to PA was determined using the 2^−ΔΔCt^ method [76].

### 4.8. Oil Red O Staining

The phthalates’ effect on lipid accumulation was assessed by Oil Red O staining according to the method described by Molonia et al. [77]. After staining, the adipogenic cultures were observed by an optical microscope and photographed. The degree of lipid droplets was determined with red pixel areas indicating fat vacuoles being divided by the total scanned area. The staining of the multiwell plate sections was quantified by ImageJ software [78] and results were expressed as the fold change against control.

### 4.9. Statistical Analysis

All the experiments were carried out in triplicate and repeated three times. Results are expressed as mean ± SD from three experiments and statistically analyzed by a one-way ANOVA test, followed by Tukey’s HSD, using the statistical software ezANOVA (https://people.cas.sc.edu/rorden/ezanova/index.html accessed on 5 April 2022). Differences in groups and treatments were considered significant for *p* < 0.05.

## 5. Conclusions

Lacking data on the potential adipogenic effects of p-phthalates, especially at environmentally relevant very low concentrations, produces a high level of uncertainty regarding their suitability as safe compounds. Given the observed effects of nanomolar concentrations of the p-phthalates studied and taking into account humans’ close and constant contact with plastics, it seems appropriate that ascertaining safe levels of TPA and DMT exposure is considered a high priority.

## Figures and Tables

**Table 1:** In silico physicochemical and pharmacokinetic parameters of TPA and DMT.

		TPA	DMT
Parameter		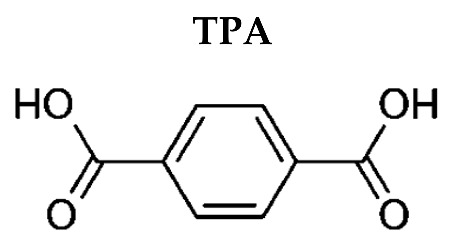	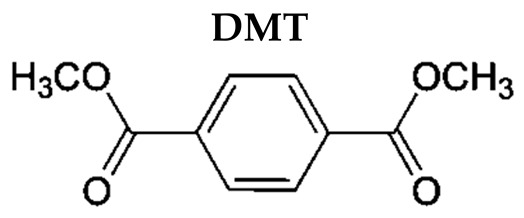
Molecular Weight		166.132	194.186
miLopP		1.76	2.28
TPSA		74.60	52.61
#Rotatable Bonds		2	2
#Acceptors		2	4
#Donors		2	0
Surface Area		68.073	81.441
Water solubility	Numeric (log mol/L)	−2.602	−1.827
Caco-2 permeability	Numeric (log Papp in 10^−6^ cm/s)	0.716	1.256
Intestinal absorption (human)	Numeric (% absorbed)	76.537	89.396
Skin Permeability	Numeric (log Kp)	−2.735	−2.512
P-glycoprotein substrate	Categorical (Yes/No)	No	No
P-glycoprotein I inhibitor	Categorical (Yes/No)	No	No
P-glycoprotein II inhibitor	Categorical (Yes/No)	No	No
VDss (human)	Numeric (log L/kg)	−2.006	−0.432
Fraction unbound (human)	Numeric (Fu)	0.556	0.368

miLopP: Molinspiration LogP; TPSA: Topological polar surface area; VDss: Steady-state volume of distribution. #: number of.

## Data Availability

The data that support the findings of this study are available upon reasonable request to the corresponding author (A.S. (Antonio Speciale)).
